# Persistent spatial working memory deficits in rats with bilateral cortical microgyria

**DOI:** 10.1186/1744-9081-4-45

**Published:** 2008-10-01

**Authors:** R Holly Fitch, Heather Breslawski, Glenn D Rosen, James J Chrobak

**Affiliations:** 1 Department of Psychology/Behavioral Neuroscience, University of Connecticut, Storrs, CT, 06269, USA; 2 Department of Genetics, Yale School of Medicine, New Haven, CT, 06510, USA; 3 Department of Neurology, Beth Israel Deaconess Medical Center, 330 Brookline Ave. Boston, MA, 02215, USA

## Abstract

**Background:**

Anomalies of cortical neuronal migration (e.g., microgyria (MG) and/or ectopias) are associated with a variety of language and cognitive deficits in human populations. In rodents, postnatal focal freezing lesions lead to the formation of cortical microgyria similar to those seen in human dyslexic brains, and also cause subsequent deficits in rapid auditory processing similar to those reported in human language impaired populations. Thus convergent findings support the ongoing study of disruptions in neuronal migration in rats as a putative model to provide insight on human language disability. Since deficits in working memory using both verbal and non-verbal tasks also characterize dyslexic populations, the present study examined the effects of neonatally induced bilateral cortical microgyria (MG) on working memory in adult male rats.

**Methods:**

A delayed match-to-sample radial water maze task, in which the goal arm was altered among eight locations on a daily basis, was used to assess working memory performance in MG (n = 8) and sham (n = 10) littermates.

**Results:**

Over a period of 60 sessions of testing (each session comprising one pre-delay sample trial, and one post-delay test trial), all rats showed learning as evidenced by a significant decrease in overall test errors. However, MG rats made significantly more errors than shams during initial testing, and this memory deficit was still evident after 60 days (12 weeks) of testing. Analyses performed on daily error patterns showed that over the course of testing, MG rats utilized a strategy similar to shams (but with less effectiveness, as indicated by more errors).

**Conclusion:**

These results indicate persistent abnormalities in the spatial working memory system in rats with induced disruptions of neocortical neuronal migration.

## Background

Abnormalities in the development of language and language-related functions (e.g., reading) occur in 5–10% of otherwise normal children [1]. In early life subjects may show expressive and/or receptive language deficits (e.g., significant delays in reaching normal language milestones), and may also exhibit fundamental defects in the ability to process rapidly changing acoustic information [2-4]. Given evidence that language-impaired populations have difficulty processing rapidly changing acoustic information in both linguistic and non-linguistic forms [5], and also have difficulty manipulating phonological components of speech [6-8], a functional relationship has been postulated between deficits in these domains [9]. Consistent with this view, research shows that non-linguistic acoustic processing scores in children as young as 6 months of age predict their subsequent language performance, both in normal and at-risk populations [10,11]. In the school-age years, language delays generally resolve to some degree, but a sizable subset of the language-disabled population goes on to exhibit specific deficits in reading (e.g., dyslexia; [12]). Dyslexics show unexpected deficits on reading performance that are not predicted from overall IQ [8]. Based on evidence that phonological awareness deficits also form a core component of dyslexia, it has been suggested that early acoustic processing difficulties may also contribute to subsequent difficulties with reading [13]. However, a substantial portion of school-age dyslexics have *no *evidence of prior language disability, and theories linking acoustic problems and subsequent reading impairments have been particularly debated for this sub-population (e.g., [14,15]).

In addition to phonological difficulties, deficits in short-term memory (STM) and working memory have been seen in dyslexics as well [16]. Typically, short-term verbal memory tasks such as word-list recall (thought to tap into the phonological STM loop posited by Baddeley [17]) have been used to show verbal STM deficits in dyslexics. In addition, evidence of higher-order deficits in the processing and manipulation of stored and new phonological information (e.g., as required by sentence processing), has been reported for dyslexics [6-8], and these latter deficits are typically described as "verbal working memory deficits." However, deficits in visuospatial STM have also occasionally been reported (see [16,18] for review), and in fact more recent findings suggest evidence of a core deficit in central executive working memory functions – which modulates both the phonological loop and visuospatial sketch pad in Baddeley's working memory model – as a key feature of dyslexia [16,18].

Despite considerable research dedicated to the study of neurophysiological underpinning of dyslexia, and intriguing evidence of anomalies in brain activation during reading tasks in dyslexics [e.g., [19,20]], a clear-cut understanding of the developmental etiology for these specific cognitive deficits remains elusive. However, Galaburda et al. [21] reported the presence of collections of improperly migrated neurons (ectopias and microgyria) in the brains of dyslexics examined *post mortem*, and more recent studies have confirmed the presence of early disruptions of cortical neuronal migration associated with reading disability [22,23]. Such findings suggest that disruptions in neuronal migration may contribute to the etiology of language-based disorders. Since prior research had shown that focal freezing lesions to the skull-cap of a postnatal day 1 (P1) rat lead to histologically comparable disruptions of cortical neuromigration, (i.e., formation of a focal region of cortical dysgenesis, or abnormally-layered microgyria; [24-26]), our lab undertook an assessment of the behavioral consequences of these cortical anomalies in a rat model.

Accumulated research has now shown that induced microgyria (produced via focal disruption of cortex in a newborn rat brain) is associated with surprisingly specific deficits in rapid acoustic processing in rodents [27-35]. Moreover, evidence suggests that these rapid auditory processing deficits are aggravated (or enhanced) by increased difficulty and complexity of task [36,37]. Thus acoustic processing studies of rodent models also suggest evidence of deficits in higher order learning and/or memory systems in subjects with focal cortical developmental disruption as seen in microgyria – prompting intriguing questions regarding the putative relationship of neuronal migration anomalies and other cognitive deficits associated with dyslexia (e.g., working memory; [16,38]). In fact, prior studies of mice with spontaneously occurring ectopias (collections of improperly migrated neurons) have revealed anomalies of memory function as well [39-43].

**Figure 1:**
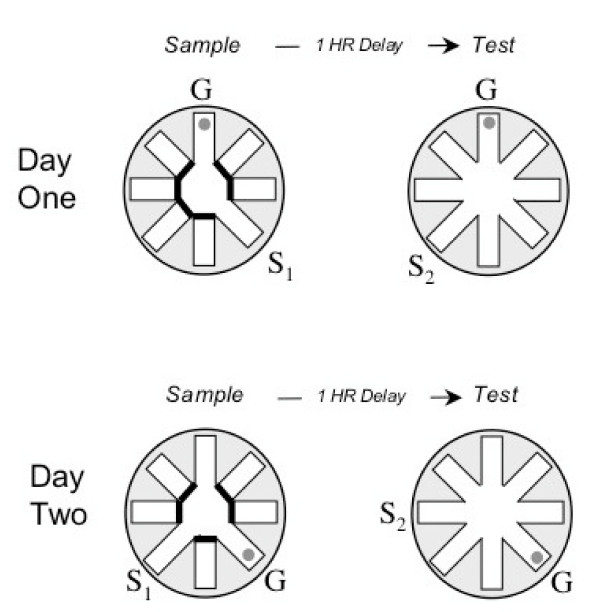
**Diagram of the delayed match to sample radial water maze task, including sample (left) and test (right) trials**. S = start (location changes), G = goal (location remains fixed). Each day rats were given one forced-choice sample-trial, with access to all but the start (S_1_) and goal (G) arm blocked. The goal arm contained a submerged platform. On the test trial (one hour later), all arms were open and a different start (S_2_) arm was used, to test memory for the spatial location of the current goal location. Only one sample and one test trial were given each day. The sequences of start arms and goal locations used each day varied systematically among forty-eight patterns that regulated the sequence of start and goal arms, the turn angles, and the relationship between the start and goal arms across trials. Exemplars for the first and second day of testing are provided (actual testing continued for 60 days).

Based on this convergent data, the current study sought to assess working spatial memory in rats that received microgyria (MG), sham surgery, or no treatment (controls). The task employed was a novel adaptation of delayed match-to-sample radial water maze paradigm, combining elements of the Morris water maze [44], the delayed match-to-sample water maze [45], and the Olton radial maze [[46]; see [47] for further task details]. The task allowed for an examination of memory for single brief (~4–20 second) experience (the location of a submerged platform in a eight arm radial water maze), with the platform location changed daily, and only one sample and one test trial each day (see Figure 1) separated by a delay interval of 1 hour. Thus the task required subjects to hold the goal-arm in STM, and/or to recall and then process the information on-line during the test trial, after the 1-hour delay. As such, this task would generally be considered a measure of working memory rather than simple short-term memory span [48]. Based on parallels between neuropathological evidence of migration anomalies (such as migrogyria) in dyslexic brains, evidence of working memory deficits in dyslexia, and prior data supporting the behavioral evaluation of an animal model of microgyria as a tool in the study of dyslexia, we predicted that MG subjects would show working memory deficits on this task. In fact, results showed that MG rats made significantly more errors on test trials, even after 60 days (12 weeks) of testing. Interestingly, the MG rats showed a similar error pattern to shams, but made more overall errors. These findings provide evidence of a weakened representation of the daily goal location in MG rats (despite using a spatial strategy comparable to shams), and suggest deficits in core working memory systems in rats with disrupted cortical neuronal migration.

## Methods

### Subjects

A total of eighteen male Sprague-Dawley rats (Charles Rivers, MA, USA) were used in these experiments (MG = 8; Sham = 10). In addition, 10 unmanipulated control male littermates were behaviorally assessed in order to confirm baseline validity of the sham treatment (and no differences between shams and unmanipulated controls were seen for any measure). All rats were pair housed, on a 12-h/12-h light-dark cycle (lights on 6 AM), in Plexiglas tubs in a temperature-controlled room. Water was available *ad libitum*. All procedures were performed in accordance with the guidelines set forth by University of Connecticut's Institutional Care and Use Committee and NIH.

### Induction of microgyria

Litters were culled to a total of 10 pups (8 males/2 females) on P1 (two female rats were always retained to normalize maternal behavior). Of the remaining 8 males, littermates were randomly assigned to one of 3 treatments – microgyric lesion induction (MG), sham control, or unmanipulated control. Briefly, pups assigned to microgyric or sham treatments were removed from the litter on P1, and subsequently received cryogenic anesthesia. Microgyric surgery followed the procedures as defined by Humphreys et al. [24]. Briefly, a cooled (-70°C) 1 mm diameter steel probe was placed on the exposed skull for 5 sec, at each of two locations – the first, approximately 2 mm lateral of the sagittal suture and 1 mm rostral of bregma, and the second, 4–5 mm caudal of the first. Following induction of the initial lesion pair (applied in a randomly determined hemisphere), an identical lesion pair was placed in the opposite hemisphere with an identical second probe kept at -70°C. Sham operated controls received similar treatment except that the probe was kept at room temperature. Pups were sutured, marked with footpad injections for identification, warmed under a lamp, and returned to the dam. Control unmanipulated male pups (and female littermates) were left with the dam during these procedures.

### Apparatus

The water maze consisted of a black Plexiglas pool (140 cm in diameter and 40 cm deep). The radial maze consisted of eight removable stainless steel corridors (painted flat black) that could be attached to a central octagonal hub. The central hub was 50 cm across with each corridor (14 cm wide), extending 36 cm to the far edge of the pool (see Figure 1). The entire apparatus was filled with cool water (22(+/- 2)°C). A removable platform constructed from black plastic (10 cm diameter) provided an escape platform that was submerged 4 cm beneath the surface of the water. The platform could be positioned at the end of any corridor, providing an escape from the water, but could not be seen by the rat. The entire apparatus was positioned in a large room with adequate spatial cues, including two empty walls, a long table, and the cage rack forming the outer boundaries (approximately 1 meter from the edge of each side of the pool). During testing, a floor lamp in the northeast corner of the room served as the light source to provide additional spatial landmark information.

### Delayed match-to-sample radial water maze training and testing

Subjects (P33) were handled for approximately five minutes each day for the week prior to training. On the initial training trial (P40), subjects were naïve to the room, as well as to the watermaze and the submerged platform. All rats were capable of navigating the corridors and using the escape platform on the initial training day. Training and testing of animals involved daily sessions (five sessions a week) comprised of two trials: a sample trial (in which the rat was guided to the platform), and a test trial in which all arms were open (see Figure 1; see [47] for more details). During the sample trial, the subject was placed in the water at the edge of a start arm. Except for the start and goal arm, all other arms were blocked at the intersection of the arm and the central hub. Each rat swam out of the corridor, navigated to the only open corridor, and mounted the submerged platform. The subject was removed immediately upon mounting the platform, gently dried with a towel and returned to the home cage on the near adjacent cage rack. Subjects took approximately 4–20 seconds to complete the sample trial. Since this study employed a delay interval of one hour, the test trial was administered 1 hour after the sample trial. During the test trial, rats were placed into a new start position. The same start position was never used between the sample and test trial, to insure navigation based on memory of spatial position rather than turn angle. Importantly, the *goal *remained in a fixed location for the sample and test trial, and during the test session, all the maze arms were open (see Figure 1). Each rat was tested each day of a five-day workweek, using a different start and goal arm each day (1 session/2 trials/day). Sequences of start arms and goal locations were varied systematically among forty-eight patterns that regulated the sequence of start and goal arms, and the relationship between the start and goal arms across trials. All arms served as start and goal arms roughly equally across each twenty-four days of testing. The goal location was restricted to arms 90 degree (2 arms) or more away from the Prior (i.e., yesterday's) goal location. Testing lasted for a total of 60 days (12 weeks), which was subsequently divided into 6 Blocks of 10 trials each.

### Dependent measures

Dependent measures included Latency to reach the platform in the sample trial (there were no errors on sample trials). Dependent measures from the test trials included: number of incorrect arm entries (Errors) during the test trial; mean Latency to reach platform (total latency divided by arms entered, i.e., an indirect index of swim speed); and the position (or type) of the First Error during the test trial (where an error occurred). The latter score (First Errors) was further broken into types as defined by: 1) Prior goal errors (entry to the arm that last served as goal location); 2) Adjacent arm errors (entry to either arm adjacent to the goal location); or 3) Other errors (random entry into any arm *other than *the correct goal arm, the prior goal arm, or an arm adjacent to the goal).

### Data analyses

Error and latency data were analyzed as a function of Treatment (2 levels; sham, MG) and Block (6 levels; each block = 10-day (2 week) series of test sessions), using repeated measures analyses of variance (ANOVA) for both order and trend, followed by paired *t*-tests for all other comparisons. The pattern of errors was further assessed using a Chi-Square analysis on the frequency distribution of Prior, Adjacent and Other arm First Errors [49]. For ease of data presentation, the First Error frequency distribution was converted to percentages, and is presented in pie chart form for the first and last Block. Analyses were conducted using SPSSX on a PC compatible computer, or using Microsoft Excel.

## Results

### Neuroanatomic assessment

**Figure 2:**
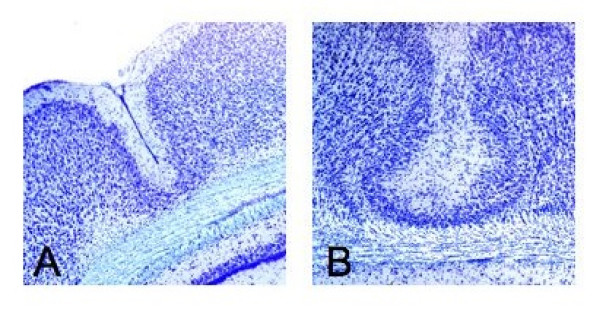
**Coronal histology, microgyric subjects**. A. Cross-section of microgyric indentation in cerebral cortex. B. Magnification of abnormal cortical layering beneath the microgyric sulcus.

Visual inspection followed by histological preparation and assessment of brain tissue from subjects in the current study confirmed the presence of microgyric malformations (visible as small indentations in the neocortical surface, and in coronal cross-section as abnormally layered regions of cortex) for all 8 microgyric subjects (Figure 2). No malformations or cortical abnormalities were seen for the 10 sham subjects.

### Errors to find the platform (test trials)

Initial statistical analyses revealed no differences between sham (n = 10) and unmanipulated control (n = 10) subjects for any measure, including overall errors (*F*'s (1,18) < 1.8), as measured by arms incorrectly entered across the 60 test trials. Based on confirmation that our sham treatment did not alter any measure as compared to unmanipulated controls, further analyses utilized comparisons between the MG and sham groups only.

**Figure 3:**
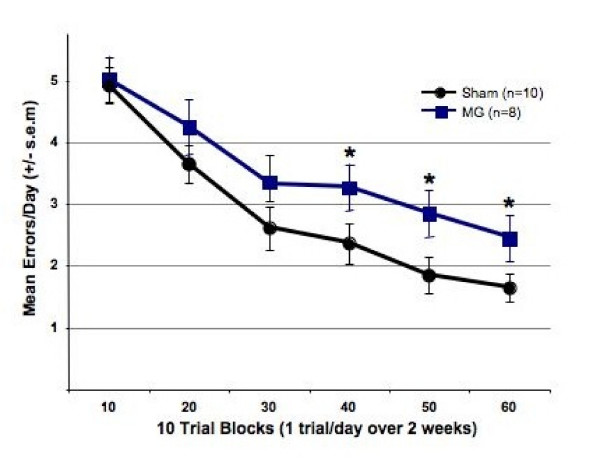
**Mean errors per day for sham and microgyric (MG) subjects over six 10-trial blocks (twelve weeks/60 days) of testing**. Overall Treatment effect on mean errors (p < 0.02) with No Treatment × Block interaction indicated that MG rats made significantly more errors than sham treated rats. Contrasts for simple-effects analysis performed within each 10 trial block indicated that that MG rats were significantly impaired compared to sham treated rats for the last three 10-trial blocks (p < 0.05).

Analyses of overall errors revealed that the presence of cortical microgyria (n = 8) impaired acquisition and performance of the delayed match-to-sample radial water maze task relative to shams. Specifically, a repeated measures ANOVA demonstrated a significant effect of Treatment [*F *(1, 16) = 8.33, *p *< 0.05, Figure 3], a significant effect of Block [*F *(5,80) = 30.46, *p *< 0.001, Figure 3], and no Block × Treatment interaction [*F *(5, 80) = 1.37, NS]. Thus, despite learning by both groups, rats with cortical microgyric disruption showed significant and sustained deficits in performance across all twelve weeks of testing. Further assessment of simple effects within each 10-trial block revealed a significant effect for Blocks 4, 5 and 6 (see Figure 3). Notably, while it has been suggested that this pattern denotes an "emergent" deficit, it is critical to point out that all rats are performing at chance levels during the first 10-trial Block. That is, since the maze has 8 available arms (including the entry and goal arms), an average of 4–5 errors is effectively chance performance to find the goal arm. Since rats are unlikely to perform worse than chance, it is therefore not possible to observe any potential Treatment effects until shams learn the task adequately (i.e., perform significantly better than chance).

### Distribution of first error types

**Figure 4:**
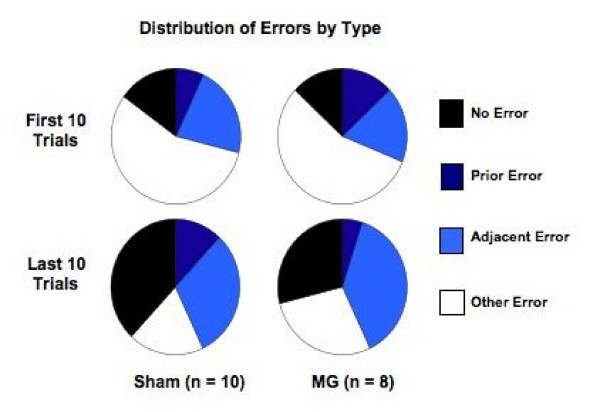
**Distribution of error types for first error made (including "No Error") in the first and last 10-trial blocks of testing**. Distributions for shams are shown on the left, MG on the right. Distributions for the first 10-trial block distribution are shown at the top, and the last 10-trial block distribution at the bottom. No differences were observed in the distribution of errors between MG and shams for any of the 10 trial Blocks. While there was an obvious increase in the number of rats making "No Error," the distribution of First Errors (when the rat made an error) also changed between the first and last 10 trials, with an increase in the percentage of rats choosing either an Adjacent arm or the Prior goal arm.

For all test trials in which an error was made, we characterized the type. Figure 4 shows the percentage of trials in which there was: 1) No error; 2) a Prior goal arm error; 3) an Adjacent goal arm error; or, 4) an error to any Other arm. Interestingly, although MG rats made significantly more errors overall compared to shams, their distribution of first error types did not differ from shams. That is, the frequency distribution of first errors during the first 10 trials (Block 1, *χ*^2 ^= 1.9, df(2), NS), and the last 10 trials (Block 6, *χ*^2 ^= 3.9, df(2), NS), indicated no Treatment difference between sham and MG rats (nor were Treatment effects found for Blocks 2, 3, 4 or 5). The First Error distribution *did*, however, change across testing, with both groups shifting towards a greater percentage of Adjacent and Prior arm errors, and fewer Other arm errors (*χ*^2 ^> 15, df(2), *p *< .001). (Noting the obvious increase in "No errors" category over training, this category was not including in the Chi-square analyses). An error to the Prior goal arm indicates proactive interference from the prior day's trial, while an error to one of the two Adjacent arms indicates weakened representation of the current goal location. Since > 50% of all first errors are to either the Prior goal arm or one of the Adjacent arms, this indicates that rats are more likely to show interference or weakening of the goal location memory when errors are made, rather than random arm selection (indicating no memory of goal location at all). The lack of group differences in the distribution of first error types indicates that the MG rats were performing the task in a similar manner to shams, even though they were making more total errors to find the correct goal location. Thus, while the spatial working memory system in MG rats may have been impaired, they seemed to be utilizing the same spatial strategy.

### Mean latencies to mount the platform (swim speed)

An ANOVA was performed on latencies to reach the platform on the sample trials, and mean latency to reach the platform on the test trials as measured by the total latency to reach platform, divided by the total number of arms entered. Thus while overall test trial latency scores were much higher for rats that made more errors, the mean latency score provided an indirect measure of swimming speed, combined with average latency to choose an arm. Results showed a significant Block effect [*F*'s (5,80) > 7.1,*p*'s < 0.01] indicating an overall reduction in latency with increased experience, but no Treatment effect [*F*'s (1,16) < 1.2, NS] nor Block × Treatment interaction [*F*'s (5,80) < 1.5, NS]. Thus, rats with cortical microgyria (MG) performed the motor component of the task, as measured by choosing and navigating (i.e., swimming) to arms, at a rate comparable to shams. This result indicates that the increase in errors for MG rats does not reflect a motor impairment or generalized delay for this group.

## Discussion

The current findings provide evidence that adult male microgyric rats exhibit impaired learning and memory on a delayed match-to-sample radial water maze task when compared to sham littermates. Moreover, evidence of higher spatial working memory errors for MG as compared to sham subjects was evident even after 60 days (12 weeks) of continuous testing – suggesting that spatial working memory deficits in rats with bilateral cortical microgyria are surprisingly persistent.

The neurophysiological underpinnings of this behavioral deficit remain unclear. However, studies have revealed that microgyric cortex is characterized in rodents by anomalous afferent and efferent connections – including homotopic and heterotopic cortico-cortical connections, as well as cortico-thalamic connections [50,51]. More recent evidence has also revealed reductions brain weight and cortical volume of microgyric rats [33], as well as reductions in callosal volume [52]. Concomitant evidence has shown regions of aberrant cortical hyperexcitability, at least in the cortical region immediately surrounding the microsulcus (e.g., [53]). Thus deleterious effects of early disruption to cortical neuronal migration appear to include robust and widespread consequences for reorganization of neural circuitry, with apparent effects on memory systems.

Interestingly, Denenberg and colleagues performed a comprehensive series of studies showing that "ectopic" mice – those with spontaneously occurring neuronal migration disorders (ectopic collections of neurons in the molecular layer) – also exhibit significant anomalies when compared to non-ectopic littermates on memory tasks. For example, ectopic BXSB/MpJ mice – a strain in which about 40–60% of mice exhibit spontaneous ectopias in and around the prefrontal cortex – display robust and replicable deficits in a water-escape version of the radial arm maze when compared to non-ectopic littermates [41,42]. Additional studies found that ectopic BXSB/MpJ mice exhibit deficits on a delayed match-to-sample version of the Morris water maze (which depends heavily on within-session or working memory [43]), and also make more working memory errors in an inverted version of the Lashley III maze [39]. Ectopic BXSB/MpJ mice also show impaired performance on two versions of a Hebb-Williams maze that emphasize working memory demands [40]. Related studies have examined the behavioral effects of early focal induced injuries to cortex in DBA/2J mice, using injury protocols resulting in cortical ectopias (P1 puncture of the cortical plate), as well as microgyric lesions. These studies reveal generalized deficits in a Morris delayed match-to-sample water maze (utilizing working memory), as well as Lashley III maze, and a non-spatial T-maze – again indicating some form of cortical disruption and re-organization that ultimately disrupts learning and memory [54]. Interestingly, deficits associated with ectopia are *not *seen in BXSB/MpJ mice for non-spatial working memory or reference memory [55,56], and in fact ectopic BXSB/MpJ mice actually perform *better *than non-ectopic littermates on reference memory tasks [39].

Notably, many of the above working memory tasks revealed that performance by ectopic and non-ectopic BXSB/MpJ mice converged after several days (sessions) of testing. For example, in the inverted Lashley III maze, working memory errors in ectopic BXSB/MpJ mice were initially worse than non-ectopic littermates, but converged with non-ectopics after about 4 days [39]. Similar effects were seen on two working memory versions of the Hebb-Williams maze, in which ectopic BXSB/MpJ mice initially showed more errors, but converged with non-ectopic littermates after 5–6 days [40]. In the radial arm maze, ectopic BXSB/MpJ mice also made significantly more working memory errors as compared to non-ectopics during acquisition (2–7 days), with ectopic deficits being less pronounced during the asymptotic phase (8–12 days; [42]; but see [41] indicating that ectopic effects on working memory errors are greater during the asymptotic phase of radial arm maze testing). Taken together, these data suggest that behavioral assessments using a more difficult (demanding) working memory task could elicit more persistent (lasting) evidence of working memory deficits in a rodent model of early cortical disruption – and the results reported here confirm that assertion. However, it must also be noted that further testing in this model might ultimately reveal convergence of performance given the continuing downward slope for both groups between Blocks 5 and 6 (Figure 3). This issue will be addressed in future studies.

In summary, convergent evidence seems to support the view that focal disruptions of neuronal migration in rodent models may relate to highly specific patterns of learning and memory deficits that could, in turn, map onto behavioral deficits that characterize human dyslexia. That is, numerous reports characterize both short-term and also working memory deficits in this population. Most frequently reported are deficits in phonological memory [6-8], but deficits on visuospatial tasks, as well as evidence of deficits in core executive working memory systems, have also been found [16,18]. Given this evidence of both verbal and visuospatial working memory deficits in dyslexics, coupled with an animal literature that generally references the maintenance of information over a 1-hour delay interval and incorporation of that information into performance of a new task trial as "working memory" [48], the current findings are presumed to reflect evidence of core working memory deficits in MG rats, which may be found to translate onto non-spatial tasks as well in future studies. Based on the critical contributions of working memory deficits to the clinical characterization of dyslexia, the current results have significant implications for understanding the neurophysiological underpinnings of dyslexia in human populations.

Finally, it is worth noting that the current results are reported in the context of concurrent rapid auditory processing deficits in similarly-treated MG rats (and these rapid auditory processing deficits also seen in mice with spontaneous ectopias as described above, [27-36,57,58]). One might question how evidence of basic acoustic processing deficits can be integrated with the evidence of working memory effects of MG reported here – particularly with regard to common underlying developmental changes in neural circuitry. Similar debates in the human literature question how higher-order disruptions of linguistic processing and working memory might relate to lower-order acoustic processing deficits, all of which have been reported in human dyslexics. One synergistic perspective derives from a co-occurrence in the same dyslexic brains that had revealed anomalies of cortex [21], of structural anomalies in auditory *thalamic *nucleus (specifically, the medial geniculate nucleus or MGN; [59]). Comparable co-occurring cortical and MGN alterations are seen in the MG rat brain [60], and behavioral evidence indicates that acoustic discrimination in MG rats is aggravated both by increasingly short duration stimuli and *also *by increasing stimulus complexity or "cognitive task load" [29,30,36,37]. Thus data exists to support both "bottom-up" and "top-down" contributions to behavioral anomalies in this model, with potential implications for the neurobiological etiology of "top-down" and "bottom-up" contributions to deficits in human dyslexics as well [61].

## Conclusion

In conclusion, we report novel evidence of spatial working memory deficits in rats with focal disruptions of neuronal migration in cortex, leading to microgyric malformations similar to those seen in human dyslexic brains. Further, we suggest that resulting deficits in working memory are consistent with the concurrent prolific literature demonstrating rapid auditory processing deficits in these same subjects, and can be reconciled via inferences that, in humans, impairments at both higher (cortical/linguistic) and lower (subcortical/sensory) processing levels may contribute to deficits seen in convergent behavioral output as ultimately measured through language and reading skills [e.g., [61]]. Future research will endeavor to assess possible links between dyslexia-risk genes known to modulate neuronal migration in cortex (e.g., *DYX1C1*) and working memory deficits in a rodent model, particularly given evidence that the latter gene has been implicated in memory deficits in human dyslexic populations [62]. Finally, future studies will examine hippocampal morphology in MG rats, as well as those with manipulations of the rat homolog gene *Dyx1c1 *[63] to assess whether various forms of developmental disruption of cortex may ultimately lead to reorganization and/or anomalous anatomy in distal structures such as hippocampus that may in turn mediate (or contribute to) the working memory deficits reported here.

## Competing interests

The authors declare that they have no competing interests.

## Authors' contributions

GDR performed the neonatal microgyric lesion surgery and participated in the drafting of the manuscript. HB performed daily behavioral testing and data collection, as well as subject perfusions. JC participated in the design of the behavioral tasks, supervised data collection, data entry, and statistical analysis, and participated in the drafting of the manuscript. RHF conceived of the study, designed and supervised all aspects of the study, and supervised the writing of the manuscript. All authors read and approved the final manuscript.
